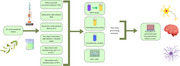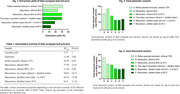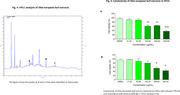# *Olea europaea* leaf extract: a natural strategy for antioxidant defense and neuroprotection

**DOI:** 10.1002/alz70859_105450

**Published:** 2025-12-25

**Authors:** Katiuscia Adam Mello, Maria Paula Faccin Huth, Murillo Orsatto Haas, Giulia Aline Führ, Aline R. Zimmer

**Affiliations:** ^1^ Federal University of Rio Grande do Sul, Porto Alegre, Rio Grande do Sul Brazil

## Abstract

**Background:**

Dementia is a multifactorial clinical condition characterized by damage and deterioration of neuronal cells, leading to cognitive decline exceeding normal aging. It has been estimated that over 10 million new cases of dementia occur every year. In this regard, it is undeniable that there is a need for new therapeutic and prevention approaches. Natural products emerge as a source to prospect new neuroprotector compounds, mainly because of their antioxidant and anti‐inflammatory potential. This study evaluated the cytotoxicity and neuroprotective potential of *Olea europaea* leaf extracts.

**Methods:**

Six extracts were obtained from dried and powdered olive leaves. The total flavonoid and phenolic content of each extract was evaluated, along with the scavenging DPPH activity. Subsequently, the extracts were analyzed using high‐performance liquid chromatography (HPLC‐DAD). The cytotoxicity and neuroprotective effects of the extracts were tested in in vitro assays using two different brain cell lines (C6 astroglial and HT‐22 neuronal cells) against inflammatory insult (LPS).

**Results:**

The extracts produced by reflux with ethanol 75% and by maceration with ethanol 80% showed the highest antioxidant potential, with EC50 of 38.29 µg/mL and 51.84 µg/mL, respectively. The extract obtained by reflux showed the highest content of flavonoids (70 mg QE/g) and a significant content of phenolics (102.72 mg GAE/g). Three flavonoids were identified in the HPLC analysis. The most promising extracts exhibited low toxicity to brain cells with IC50 values between 321.9 and 365.6 µg/mL in HT‐22 neuronal cells and 335.0 and 208.0 µg/mL in C6 astroglial. Regarding neuroprotection, ethanol 75% extract protected the C6 astroglial from neuroinflammatory insult at 31 µg/mL.

**Conclusion:**

The extracts, particularly those obtained with ethanol and heating, showed excellent antioxidant properties and potential for neuroinflammatory protection. These extracts may serve as a basis for designing new molecules and strategies for treating and preventing neurodegenerative diseases.